# Simultaneous management of kidney stones and ureteral strictures using tip-bendable ureteral access sheath assisted transabdominal approach combined with laparoscopic upper urinary tract reconstruction

**DOI:** 10.1186/s12894-025-01988-0

**Published:** 2025-11-17

**Authors:** Yuli Zhao, Qi Zhong, Zhiwen Wang, Xin Huang, Jin Kuang, Fei Li, Xiaolin Deng

**Affiliations:** 1 Medical Imaging Center, Ganzhou People’s Hospital, Ganzhou, Jiangxi 341000 China; 2 Department of Urology, Ganzhou People’s Hospital, Ganzhou, Jiangxi 341000 China; 3 Department of Urology, Nanfang Hospital, Southern Medical University, Guangzhou, Guangdong PR China

**Keywords:** Ureteral strictures, Kidney stones, Ureteral access sheath, Laparoscopic upper urinary tract reconstruction

## Abstract

**Purpose:**

The treatment of ureteral strictures combined with kidney stones often necessitates staged surgical intervention, increasing the patient burden and leading to the inefficient use of medical resources. This study aimed to evaluate the efficacy of simultaneous treatment of ureteral strictures and kidney stones using tip-bendable ureteral access sheath (UAS)-assisted transabdominal flexible ureteroscopic lithotripsy (FUL) combined with laparoscopic upper urinary tract reconstruction, potentially providing a safer and more effective therapeutic option.

**Methods:**

We retrospectively reviewed data from 21 patients with ureteral strictures and kidney stones who underwent tip-bendable UAS-assisted transabdominal FUL combined with laparoscopic upper urinary tract reconstruction between January 2023 and June 2024. We analyzed the stone-free rate (SFR), operative time, postoperative hospital stay, complications, changes in laboratory parameters, and hydronephrosis.

**Results:**

Complete stone clearance was achieved in 19 (90.5%) patients. The mean stone size was 20.0 ± 4.7 mm, and the mean operative time was 136.9 ± 23.8 min. Postoperative renal function significantly improved with reduced serum creatinine levels (*P* = 0.024) and an increased estimated glomerular filtration rate (*P* < 0.001). Hydronephrosis was notably reduced, with only two patients exhibiting mild dilation. Furthermore, only minor Grade I complications occurred, including fever and nausea.

**Conclusions:**

Tip-bendable UAS-assisted transabdominal FUL combined with laparoscopic upper urinary tract reconstruction via the transabdominal approach is an effective and safe strategy for simultaneously managing ureteral strictures and kidney stones. This approach resulted in a high SFR, improved renal function, and minimal complications.

**Supplementary Information:**

The online version contains supplementary material available at 10.1186/s12894-025-01988-0.

## Introduction

Ureteral stones are a common condition in urology, with an incidence rate of 5–12% [1]. Ureteroscopy with holmium laser lithotripsy is the first-line treatment for ureteral stones [2], with postoperative ureteral strictures occurring in 0.1–5.8% of cases [3, 4]. Recently, the incidence of iatrogenic ureteral injuries has increased, largely due to the increasing number of endourological interventions and pelvic surgeries in gynecology and gastroenterology [5, 6]. Laparoscopic or robot-assisted upper urinary tract reconstruction has become an effective treatment option for ureteral strictures, offering a high success rate, favorable long-term outcomes, and a low risk of complications [7, 8]. However, owing to prolonged ureteral obstruction, some patients develop ipsilateral kidney stones, often requiring staged procedures to treat both the stones and strictures. This can place significant psychological and financial burdens on patients and result in the inefficient use of medical resources.

In 1983, Sharma et al. [9] were the first to report the simultaneous treatment of kidney stones and ureteral strictures in 15 patients using pyelolithotomy combined with calycoureteroplasty, which yielded favorable long-term outcomes. More recently, several researchers have successfully performed upper urinary tract reconstruction combined with flexible ureteroscopic lithotripsy (FUL) [10] or percutaneous nephrolithotomy (PCNL) [11], highlighting the efficacy of minimally invasive approaches for treating both conditions simultaneously. We previously attempted to use a flexible ureteroscope and stone basket to remove renal stones during upper urinary tract reconstruction by entering the renal pelvis through the proximal severed ureter to treat kidney stones; however, limited maneuverability, poor visualization, and low stone extraction efficiency hindered satisfactory results.

In 2022, Chen et al. [12] introduced a novel tip-bendable ureteral access sheath (UAS) that incorporated a bendable tip and proximal suction channel into the traditional UAS design. When used with a flexible ureteroscope, the tip-bendable UAS passively bends to follow the ureteroscope into the renal calyces and, combined with negative-pressure suction, enables real-time removal of stone fragments. Previous studies have demonstrated that the use of a tip-bendable UAS during retrograde intrarenal surgery (RIRS) can significantly improve the stone-free rate (SFR) and reduce the risk of pressure-related complications.

We report the initial exploration of the simultaneous management of kidney stones and ureteral strictures using tip-bendable UAS-assisted transabdominal FUL combined with laparoscopic upper urinary tract reconstruction.

## Materials and methods

### Patients

We retrospectively collected data from patients treated between January 2023 and June 2024 at the Department of Urology at Ganzhou People’s Hospital, China. These patients underwent tip-bendable UAS-assisted transabdominal FUL combined with laparoscopic upper urinary tract reconstruction for the simultaneous treatment of ureteral strictures and kidney stones. The inclusion criteria were as follows: (1) patients aged 18–75 years, (2) diagnosis of ureteropelvic junction obstruction or upper ureteral strictures, and (3) presence of a single renal stone with a maximum diameter or multiple stones with a cumulative diameter of < 2 cm. The exclusion criteria were as follows: (1) previous laparoscopic or open surgery in the operative region, (2) severe cardiopulmonary insufficiency, (3) coagulation disorders or significant liver or kidney dysfunction, and (4) intraoperative findings of pyuria. All data were sourced from the Hospital Information System of Ganzhou People’s Hospital, and privacy-sensitive patient information was removed prior to analysis.

The location and length of the ureteral strictures were assessed using enhanced urinary computed tomography (CT), intravenous urography, or retrograde urography. Stone size was evaluated using CT, with the maximum diameter recorded as the stone burden. Ureteral stents were routinely removed 2 months postoperatively, and the SFR and ureteral stricture outcomes were evaluated 1 month after stent removal (3 months postoperatively) using urinary CT and ultrasound. Preoperative and postoperative hydronephrosis were compared by measuring the renal pelvic anteroposterior diameter (APD) and applying the Society for Fetal Urology (SFU) grading system.

Broad-spectrum antibiotics were administered preoperatively to prevent infections. In patients with positive preoperative urine cultures, sensitive antibiotics were administered until a negative urine culture was obtained before surgery. Perioperative complications were recorded and classified using the Clavien–Dindo classification.

This study adhered to the 2013 revision of the World Medical Association Declaration of Helsinki and was approved by the Ethics Committee of Ganzhou People’s Hospital (TY-ZKY2024-010–21).All patients voluntarily participated in this study and signed informed consent. All patients provided written informed consent prior to surgery.

### Surgical technique

**Fig. 1:**
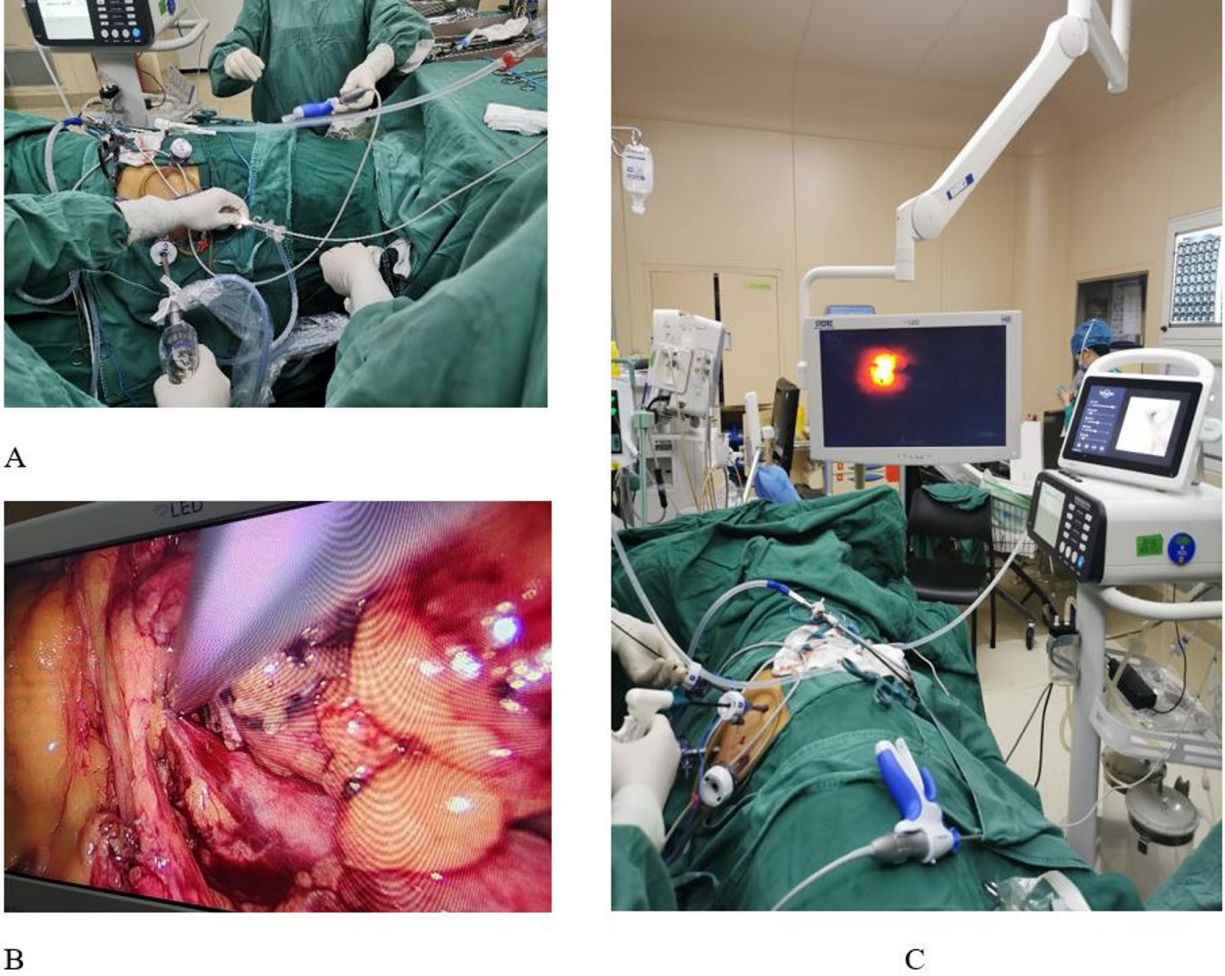
Intraoperative image. **A** Tip-Bendable UAS introduced through a 5-mm laparoscopic port; **B ** UAS inserted to renal through incision made in the renal pelvis;**C** Ureteral stricture identified by flexible ureteroscope and laparoscope

Following successful general anesthesia, the patient was placed in the lateral decubitus position, and the surgical field was disinfected and draped in a standard manner. Following a routine procedure, three laparoscopic ports were inserted transperitoneally in the upper urinary tract region. After establishing a laparoscopic working space by insufflating with carbon dioxide, the lateral peritoneum was incised using an ultrasonic scalpel, and the surrounding tissues were dissected to expose the kidney and upper ureter. The renal pelvis and the upper ureter were mobilized. A 5-mm incision was made in the renal pelvis, and a tip-bendable UAS (Huamei, Zhangjiagang, China) was introduced through a 5-mm laparoscopic port and advanced into the renal pelvis under laparoscopic guidance. A flexible ureteroscope (Ruipai, Guangdong, China) was passed through the tip-bendable UAS into the renal pelvis and calyces. After identifying the stones, a 275-µm fiber was connected to a holmium laser (Raykeen, Shanghai, China), which was passed through the ureteroscope to fragment the stones. The stone fragments were subsequently extracted by suction. Once the stones were completely removed, the flexible ureteroscope and tip-bendable UAS were implanted into the distal ureter. Ureteral strictures were identified directly using flexible ureteroscopy and adjusting the light intensity of the laparoscope. The ureteral stricture was identified and fully mobilized (Fig. 1). After excision of the stenotic segment and adjacent scar tissue, spatulation was performed on both ends of the ureter 180° apart. End-to-end anastomosis was performed using 3 − 0 Vicryl sutures, with a 6-Fr double-J stent placed prior to completing the anastomosis. A routine abdominal drainage tube was inserted. The renal pelvic incision was closed using 4 − 0 Vicryl sutures.

### Statistical analysis

Data were analyzed using SPSS version 29.0. Normally distributed continuous variables were expressed as mean ± standard deviation (x̄ ± s), while ordinal and non-normally distributed continuous variables were presented as median (Q1, Q3). Categorical data were presented as n (%). The Wilcoxon signed-rank test was used for paired ordinal data or non-normally distributed continuous variables, while the paired t-test was used for normally distributed continuous variables. Statistical significance was set at *P* < 0.05.

## Results

The patients’ perioperative clinical data are summarized in Table 1. A total of 21 patients were included in this study (mean age, 48.7 ± 15.9 years; males: 12, females: 9). The mean preoperative stone size was 20.0 ± 4.7 mm, with an average stone density of 1015.7 ± 309.8 Hounsfield units (HU). All patients presented with solitary stones, with their locations distributed as follows: three stones in the upper calyx, four in the middle calyx, nine in the lower calyx, and five in the renal pelvis. Regarding upper urinary tract obstruction, 15 patients had proximal ureteral strictures, while six had ureteropelvic junction obstruction, with a mean stricture length of 10.9 ± 4.3 mm. Complete stone clearance was achieved in 19 of the 21 patients, resulting in an SFR of 90.5%. The mean operative time was 136.9 ± 23.8 min, and the average postoperative hospital stay was 7.9 ± 2.8 days. Only grade I perioperative complications were observed, including fever in two patients and nausea in three patients. No grade II–V complications were reported.

**Table 1:** Demographics and baseline characteristics of the patients

Variables	Values
No. of patients	21
Age, years	48.7 ± 15.9
Sex	
Male	12 (57.1%)
Female	9 (42.9%)
Stone distribution	
Upper calyx	3 (14.3%)
Middle calyx	4 (19.0%)
Lower calyx	9 (42.9%)
Renal pelvis	5 (23.8%)
Stone size, mm	20.0 ± 4.7
Stone density, HU	1015.7 ± 309.8
Location of stricture	
UPJO	6 (28.6%)
Upper ureter	15 (71.4%)
Length of stricture, mm	10.9 ± 4.3
SFR, %	90.5% (19/21)
Operative time, min	136.9 ± 23.8
Postoperative hospital stay, days	7.9 ± 2.8
Clavien–Dindo classification grading system	
Grade I	
Fever (≥ 37.3℃)	2 (9.5%)
Nausea	3 (14.3%)
Grade II–V	None

*UPJO* Ureteropelvic junction obstruction, *SFR* Stone-free rate

Regarding the perioperative indicators, significant differences were observed between preoperative and postoperative hemoglobin levels (137.0 ± 20.6 g/L vs. 127.6 ± 16.1 g/L, *P* < 0.001), serum creatinine levels [90.5 (72.4, 117.1) µmol/L vs. 81.3 (62.3, 93.5) µmol/L, *P* = 0.024], and the estimated glomerular filtration rate (eGFR) (85.7 ± 29.1 mL/min/1.73 m² vs. 96.0 ± 29.2 mL/min/1.73 m², *P* < 0.001). One month after the removal of the double-J stent, follow-up evaluations revealed a significant improvement in hydronephrosis. The median preoperative renal pelvic APD was 36.7 (32.2, 47.1) mm, and postoperatively, only two patients exhibited mild renal pelvic dilation. There was a statistically significant difference in the renal pelvic APD between the preoperative and postoperative measurements (*P* < 0.001). Furthermore, the postoperative SFU grade improved significantly compared with the preoperative levels (*P* < 0.001). Postoperatively, only two patients had Grade 1 hydronephrosis, with renal pelvic APDs of 11.6 mm and 13.5 mm, respectively, both showing marked improvement from their preoperative condition (Table 2).

**Table 2:** Comparison of perioperative data

Variables	Preoperative	Postoperative	*P*
Hemoglobin, g/L	137.0 ± 20.6	127.6 ± 16.1	< 0.001
Serum creatinine, µmol/L	90.5 (72.4, 117.1)	81.3 (62.3, 93.5)	0.024
eGFR, mL/min/1.73 m²	85.7 ± 29.1	96.0 ± 29.2	< 0.001
Renal pelvic APD, mm	36.7 (32.2, 47.1)	0 (0, 0)	< 0.001
SFU grade, n (%)			< 0.001
Grade 0	0 (0.0%)	0 (0.0%)	
Grade 1	1 (4.8%)	2 (9.5%)	
Grade 2	4 (19.0%)	0 (0.0%)	
Grade 3	8 (38.1%)	0 (0.0%)	
Grade 4	8 (38.1%)	0 (0.0%)	

*eGFR* Estimated glomerular filtration rate, *APD* Anteroposterior diameter, *SFU* Society for Fetal Urology

## Discussion

This study explored an innovative tip-bendable UAS-assisted transabdominal FUL combined with laparoscopic upper urinary tract reconstruction to simultaneously treat ureteral strictures and renal stones. Our preliminary results demonstrated the efficacy and safety of this method, offering a promising therapeutic option for patients with concurrent conditions. To our knowledge, this is the first report of this technique.

Our study achieved a 90.5% SFR, with a mean operative time of 136.9 ± 23.8 min and a postoperative hospital stay of 7.9 ± 2.8 days. Significant improvements in hydronephrosis (renal pelvic APD and SFU grade) and renal function (serum creatinine and eGFR) were observed, confirming the efficacy of the procedure without compromising reconstructive success. The technique demonstrated safety, with no Clavien–Dindo Grade II or higher complications. Although the changes in hemoglobin levels were statistically significant, they were clinically insignificant and were likely due to surgical trauma.

Recently, the incidence of postoperative ureteral strictures has risen with the increased adoption of minimally invasive and endoscopic surgical techniques [5, 6]. Laparoscopic or robot-assisted upper urinary tract reconstruction remains the optimal approach for patients with poor outcomes after endoscopic treatment. Many patients with ureteral strictures present with ipsilateral renal stones, either due to iatrogenic injury during stone treatment or secondary stones resulting from the long-term use of ureteral stents [13]. In clinical practice, these cases are typically managed with staged surgeries for ureteral strictures and renal stones, although some researchers have explored simultaneous treatment of both conditions.

Since Sharma et al. first reported the simultaneous management of renal stones and ureteral strictures with pyelolithotomy and calycoureteroplasty in 1983 [9]; however, there has been limited progress in this area. Approximately 20 years later, Ramakumar et al. [14] introduced laparoscopic pyeloplasty combined with pyelolithotomy and achieved outcomes comparable to those of open surgery. More recent reports describe similar techniques, such as upper urinary tract reconstruction combined with RIRS or PCNL; however, these are often limited to small case studies [10, 11]. Earlier methods, such as open pyelolithotomy combined with calyco-ureteroplasty, were effective for larger stones but resulted in significant trauma and extended recovery times. Laparoscopic surgery mitigates some of these drawbacks but still causes residual kidney stones and considerable renal damage. More recently, robot-assisted reconstructions combined with PCNL have significantly reduced surgical trauma but may increase the risk of bleeding and infection inherent to PCNL [15].

In contrast, our method strikes a favorable balance between invasiveness, operational flexibility, cost-effectiveness, and the learning curve. The tip-bendable UAS improves on the traditional UAS by incorporating a flexible distal end and proximal negative-pressure interface [12]. This allows passive bending with a flexible ureteroscope, enabling the sheath to reach the calyx where the stone is located. Under negative pressure suction, stone fragments were completely extracted compared to the stone basket technique. Several studies on the tip-bendable UAS in RIRS have demonstrated its ability to improve the SFR and reduce complications [16–18]. In our study, the tip-bendable UAS was introduced through the laparoscopic port into the renal pelvis for flexible ureteroscopic laser lithotripsy, combining the advantages of flexible ureteroscopy and suction technology but avoiding the positioning of transurethral FUL. This mechanism enhances stone clearance and reduces the risk of complications associated with elevated intrapelvic pressure. The innovation of this study lies in the introduction of a tip-bendable UAS through a laparoscopic port, combining the precision of laparoscopic techniques with the flexibility of a flexible ureteroscope in the renal pelvis and calyces. This dual approach not only improves the SFR but also precisely identifies ureteral stenosis, thereby improving the effectiveness of ureteral reconstruction. Notably, no severe complications (Clavien–Dindo Grade II–V) were observed, and only a small number of patients experienced mild Grade I complications, indicating a high safety profile. Nevertheless, potential complications must be monitored, and further studies are needed to assess the long-term safety of this technique.

Despite these encouraging results, this study had some limitations. The relatively small sample size limits the generalizability of our findings, and the short follow-up period precludes the assessment of long-term outcomes. As this was a single-center study, there was a risk of selection bias. Moreover, the lack of direct comparison with traditional staged surgery makes it difficult to directly quantify the advantages and disadvantages of the two approaches. We recommend larger multicenter randomized controlled trials with extended follow-ups to assess the long-term outcomes, particularly regarding renal function. Future research should explore the application of this technique in more complex cases, such as multiple stones or longer ureteral strictures.

## Conclusion

In conclusion, our preliminary study demonstrated that tip-bendable UAS-assisted transabdominal FUL combined with laparoscopic upper urinary tract reconstruction is a safe and effective approach for the simultaneous treatment of ureteral strictures and renal stones. This innovative, minimally invasive technique offers a valuable new treatment option for patients with smaller stone burdens and less complex ureteral strictures, potentially improving patient outcomes and optimizing resource utilization. However, careful patient selection, surgeon experience, and the resources available at medical facilities remain critical factors in choosing the most appropriate treatment. Although the initial results are promising, further large-scale studies are necessary to validate the long-term clinical value and outcomes of this approach.

## Supplementary Information

Supplementary Material 1.

## Data Availability

Data is provided within supplementary information files.
